# Clinical and economic impacts of a clinical pathway-based single disease payment policy on hysterectomy for uterine fibroids: a decade-long interrupted time series analysis

**DOI:** 10.3389/fpubh.2026.1751890

**Published:** 2026-03-18

**Authors:** Hui Zheng, Zelong Li, Hang Lin, Huan Yi, Xiangqin Zheng

**Affiliations:** 1 College of Clinical Medicine for Obstetrics & Gynecology and Pediatrics, Fujian Medical University, Fuzhou, China; 2 Fujian Maternity and Child Health Hospital, Fuzhou, China

**Keywords:** China, effect evaluation, interrupted time series analysis, medical expenses, single disease payment

## Abstract

**Objective:**

In 2017, China introduced clinical pathway-based single disease payment (CP-SDP) to curb healthcare costs, yet its impact is uncertain. Using uterine fibroids as a case, we evaluated its effectiveness.

**Methods:**

We analyzed 4,727 hysterectomy cases for uterine fibroids from a tertiary hospital in Fujian Province between January 2014 and December 2023. Using an interrupted time series (ITS) with August 2017 as the intervention point, we fitted a segmented regression model to estimate immediate level changes and post-intervention trends. The impact of CP-SDP was evaluated across structure, process, and clinical outcome domains.

**Results:**

The utilization of laparoscopic surgery significantly increased from 45.1 to 75.5%, accompanied by 3.51 days reduction in average hospital stay. Significant changes had occurred in the cost structure: technical service fees (e.g., pathological diagnostics [+32.4%] and imaging [+7.3%]) increased, whereas consumable costs (e.g., antibacterial drugs [−48.5%] and disposable supplies [−19.5%]) decreased significantly. Hospitalization expenses decreased by 2.7%, while the postoperative complication rate decreased from 7.8 to 4.9%. ITS analysis showed that after the policy, hospitalization expenses immediately decreased by 2265.51 ¥ (13.28%) and continued to decrease. The long-term trend of out of pocket expenses had shifted from increasing to decreasing, and the proportion of medical insurance reimbursement had shifted from decreasing to increasing. In 2023, out-of-pocket expenses had reached their lowest level, whereas the reimbursement ratio had peaked.

**Conclusion:**

CP-SDP appears to control costs while improving care quality by promoting minimally invasive procedures and optimizing cost allocation. This approach substantially reduces patients’ long-term financial burden.

## 1 Background

The continuous rise in global healthcare costs and the challenges of resource utilization efficiency pose severe problems for healthcare systems worldwide (1). In China, the expenditure of the national basic medical insurance fund has been continuously increasing with a year-on-year growth of 5.2% (2). Payment methods profoundly influence the behavior of healthcare providers (3). Post-payment methods (such as fee-for-service) are often associated with excessive medical care, while pre-payment methods (such as capitation payment, single disease payment, diagnosis-related groups (DRGs) payment, and diagnosis-intervention packet (DIP) payment) aim to incentivize cost control (4).

To address the drawbacks of fee-for-service payment, China has actively promoted the reform of medical insurance payment methods. However, limited by factors such as information infrastructure, case management capabilities, and medical record quality, the direct introduction of complex payment models like DRGs or DIP faces challenges. Against this backdrop, a domestically adjusted clinical pathway-based single disease payment (CP-based SDP) system has emerged. CP-based SDP sets standardized clinical pathways and fixed payment amounts for specific diseases, using “quota pre-payment” to incentivize medical institutions to proactively optimize cost structures and standardize diagnostic and treatment behaviors (5). This system was implemented nationwide in 2017, with its core elements including disease identification and coding based on the International Classification of Diseases (ICD), as well as cost accounting and payment standard formulation based on historical data or clinical pathways.

Existing studies have shown that CP-based SDP can effectively reduce hospitalization costs for target diseases shortly after the introduction of CP-based SDP. For example, the case-based payment reform in Anhui Province has observed significant decreases in hospitalization costs, shortening of the average length of stay, and a notable reduction in the proportion of drug costs (6). Similarly, in Fujian Province, substantial reductions in hospitalization costs for type 2 diabetes and lacunar infarction have been found, along with a decrease in infection rates (7). Reductions in the use of unnecessary drugs and shortening of the average length of stay have also been observed in patients undergoing cataract surgery (8). These findings confirm the potential of SDP in cost control. However, most previous studies are cross-sectional and fail to deeply explore the dynamic evolution of policy effects, including the immediate effects and long-term trends (9, 10). Additionally, most studies rely on single outcome (such as length of stay, infection rate), lacking a systematic evaluation of medical resource allocation, diagnostic and treatment processes, and patient outcomes based on Donabedian’s three-dimensional quality theory (structure-process-outcome) (11).

Uterine fibroids, as the most common benign tumor in women of childbearing age, have an accumulated incidence rate of 70–80% in the 35–50 age group (12). Surgical treatment (especially hysterectomy) constitutes a significant point of health resource consumption (13, 14). Different approaches to total hysterectomy (laparoscopic, abdominal, vaginal) differ significantly in costs, length of hospital stay, and technical requirements (15). This cost gradient between surgical procedures provides an ideal scenario for observing the impact of payment policies on clinical pathway selection. It is worth noting that the development of laparoscopic technology in China coincided with the implementation period of the SDP policy (16), which creates an opportunity to explore the interaction mechanism between cost control policies and the diffusion of technological innovation.

This hospital-based study used data from 2014 to 2023 on patients who underwent hysterectomy for uterine fibroids to reveal the immediate effects and long-term trends of policy intervention on a range of outcomes. Considering the CP-based SDP policy implemented in August 2017 as the intervention point, we aimed systematically evaluate the comprehensive impact of the policy in the dimensions of structure (such as resource allocation, surgical procedure selection), process (such as service intensity, cost composition), and outcome (such as clinical outcomes, economic burden).

## 2 Methods

This study employed an interrupted time series (ITS) design to evaluate the immediate impact and long-term trend of the implementation of CP-based SDP policy in August 2017 on patients undergoing hysterectomy for uterine fibroids. Clinical data were collected from Fujian Maternity and Child Health Hospital, the largest obstetrics and gynecology hospital in Fujian Province with leading volumes of gynecological surgeries in Fujian Province, between January 2014 and December 2023.

### 2.1 Theoretical framework

A “structure-process-outcome” dynamic analytical framework was constructed by integrating Donabedian’s tripartite quality model and Roemer’s Law of health service supply (17). Donabedian’s model assesses quality improvements across three dimensions: structure (patient characteristics, medical resource allocation), process (service intensity, treatment workflows), and outcome (clinical results, cost-effectiveness). Roemer’s Law was used to analyze how healthcare institutions under prospective payment systems might adjust service intensity (e.g., surgical approach selection, diagnostic pathway optimization) to balance cost control and quality maintenance.

### 2.2 Data source

**Figure 1:**
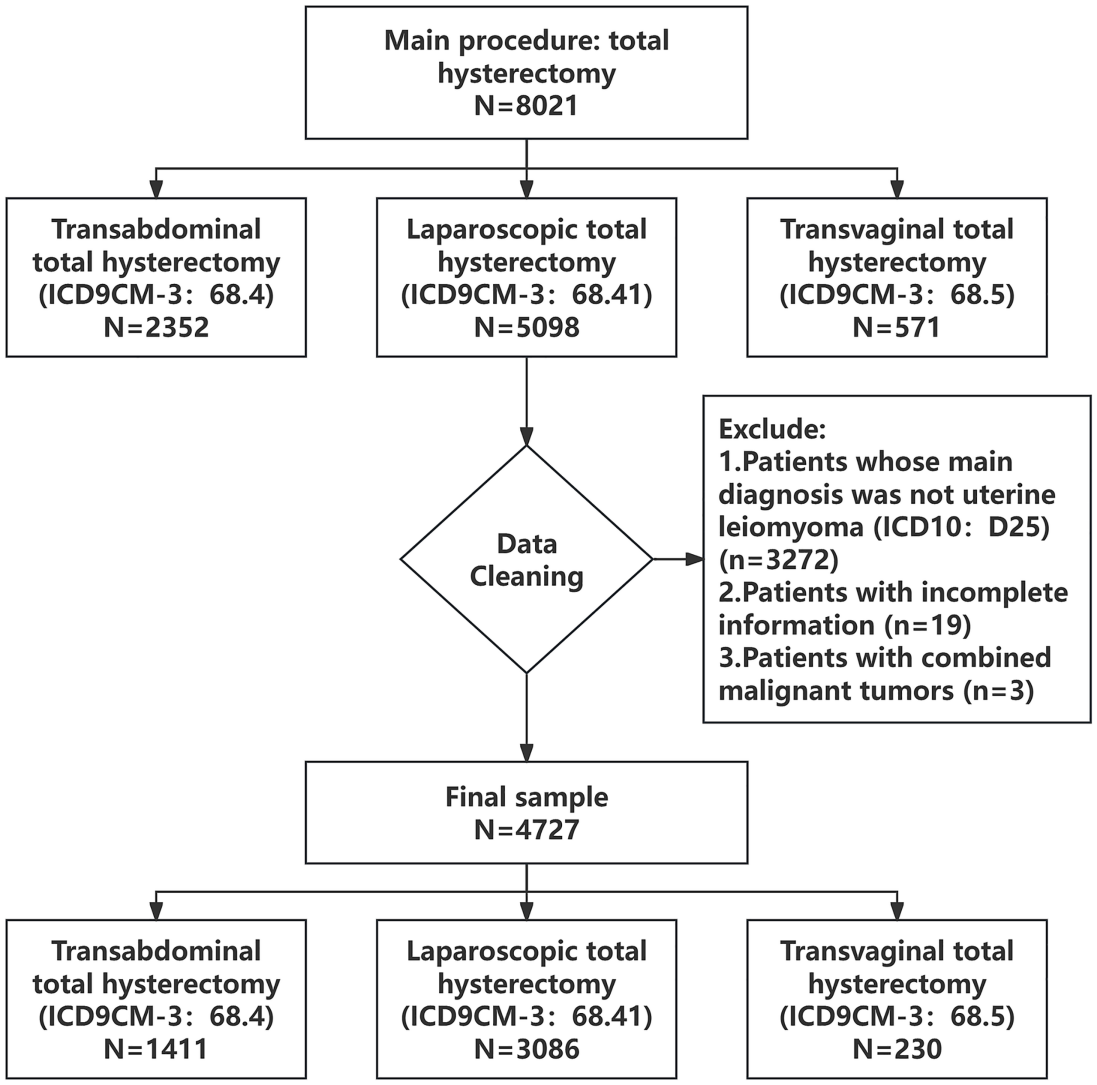
Data processing flow chart.

Data were extracted from the hospital’s Health Information System (HIS). Inclusion criteria were: (1) admission between January 2014 and December 2023 with a primary diagnosis of uterine fibroids (ICD10: D25); (2) receipt of total or subtotal hysterectomy (ICD9CM-3: abdominal 68.4; laparoscopic 68.41; vaginal 68.5). Exclusion criteria included concurrent malignant tumors or incomplete data. After applying inclusion and exclusion criteria, 4,727 consecutive cases were included (Figure 1). Notably, throughout the study period, all hysterectomies for uterine fibroids at the institution were conducted as inpatient procedures; no day surgery or outpatient surgical programs were implemented for this specific condition, ensuring the continuity and comparability of the longitudinal data.

### 2.3 Variable definition

The following variables were selected from HIS, which included age, body mass index (BMI), drug allergy, average length of stay (days), operation time (hours), hospitalization expense, out of pocket expense, medical insurance expense, general medical service fee, general treatment operation fee, nursing fee, pathological diagnosis fee, laboratory diagnostic fee, imaging diagnosis fee, surgical treatment fee, drug treatment fee, cost of antibacterial drugs, cost of disposable medical materials, admission route (emergency, outpatient, transfer from other medical institutions, others), surgical approach (abdominal, laparoscopic, vaginal), presence of surgical complications, nosocomial infection, emergency surgery, reoperation in the operating room, transfer out of and return to intensive care unit (ICU), blood transfusion (no transfusion, <5 units or 1,000 mL, ≥5 units or 1,000 mL), diabetes, hypertension, and thrombosis. All expense data were adjusted to 2013 comparable prices using the annual Consumer Price Index (CPI) index published by the National Bureau of Statistics (18). Based on Donabedian’s framework, the above variables were classified to construct a three-level index system (Table 1).

**Table 1:** Variable classification.

Dimension	Variable category	Specific variables
Structural dimension	Patient characteristics	Age, BMI, Diabetes, Hypertension
	Medical resource allocation	Surgical approach, Emergency operation
Process dimension	Service intensity	General medical service fee, General treatment operation fee, Nursing fee, Pathological diagnosis fee, Laboratory diagnostic fee, Imaging diagnosis fee, Surgical treatment fee, Drug treatment fee, Cost of antibacterial drugs, Cost of disposable medical materials
	Treatment process	Admission route, Operative time, Average length of stay
Result dimension	Economic results	Hospitalization expense, Out of pocket expense, Medical insurance expense
	Clinical outcomes	Surgical complications, Thrombosis, Blood transfusion, Nosocomial infection, Return to the operating room for further surgery, Transfer out of ICU and then return to ICU

BMI, body mass index; ICU, intensive care unit.

### 2.4 Statistical analysis

Baseline characteristics and outcome indicators were compared between the pre-policy (*n* = 1,587) and post-policy (*n* = 3,140) groups. Continuous variables were described as mean ± standard deviation and compared using independent samples *t*-tests (for normal distribution) or Mann–Whitney U tests (for non-normal distribution). Categorical variables were reported as frequencies (percentages) and compared using chi-squared tests or Fisher’s exact tests.

Interrupted Time Series Analysis (ITS) with piecewise regression models was used to evaluate policy impacts on continuous time-series data, assessing both level shifts and trend changes (19). The model was specified as: Y_t_ = β_0_ + β_1_T_t_ + β_2_X_t_ + β_3_T_t_X_t_ + ε_t_, where Y_t_ is the outcome variable, T_t_ is the time ordinal, X_t_ is a policy intervention dummy variable (0 = pre-policy, 1 = post-policy), β_0_ represents the baseline level at the start, β_1_ reflects the pre-policy trend (slope), β_2_ indicates the immediate level change at policy implementation, β_3_ denotes the change in trend post-policy relative to pre-policy, and ε_t_ is the error term. Subgroup ITS analyses by surgical approach (laparoscopic, abdominal, vaginal) were conducted to explore heterogeneity in policy effects. All ITS results were subjected to Durbin Watson (DW) test and adjusted using the Prais Winsten method to reduce autocorrelation effects.

Statistical analyses were performed using R 4.2.1, with statistical significance defined as *p* < 0.05.

### 2.5 Ethics, consent, and permissions

This research proposal has been reviewed and approved by the Ethics Committee of Fujian Maternity and Child Health Hospital (Approval Number: 2025KY113). Due to the use of retrospective anonymous data, the requirement for patient informed consent has been waived.

## 3 Results

### 3.1 Multidimensional changes after policy implementation

Apart from age, the demographic and clinical characteristics, such as body mass index (BMI) and complications (diabetes, hypertension), did not shown difference before and after the implementation of policy. After the implementation of the policy, the proportion of laparoscopic surgery in the structural dimension jumped from 45.1 to 75.5% (*p* = 0.014). The proportion of elective surgeries increased from 86.1 to 91.0% (*p* < 0.001). The process dimension showed that the average length of hospital stay was shortened by 3.51 days (13.50 days vs. 9.99 days, p < 0.001). At the same time, there has been a significant adjustment in the cost structure. Technical service fees such as pathology diagnosis fees (increased by 32.4%), imaging diagnosis fees (increased by 7.3%), and surgical treatment fees (increased by 2.8%) had increased (*p* < 0.001). Consumable expenses such as drug treatment fee (decreased by 25.4%) and cost of disposable medical materials (decreased by 19.5%) decreased (*p* < 0.001). The hospitalization expenses decreased by 2.7% (*p* < 0.001), of which medical insurance expense decreased by 18.3% (p < 0.001) and the mean out of pocket expense increased by 3.5% (*p* = 0.018) (The results of other variables are shown in Table 2). The incidence of complications decreased from 7.8 to 4.9% (*p* < 0.001), while the Nosocomial infection rate decreased from 6.2 to 2.9% (*p* < 0.001). But the proportion of large blood transfusions increased from 0.8 to 2.1% (*p* = 0.003).

**Table 2:** Comparison of baseline characteristics and hospitalization indicators before and after policies (China, 2014–2023).

Variables	Before policy (*N* = 1,587)	After policy (*N* = 3,140)	*p*-value
	Mean (SD)	Mean (SD)	
Age (year)	47.062 (0.115)	48.463 (0.090)	<0.001
BMI (kg/m^2^)	23.246 (0.075)	24.320 (0.829)	0.358
Average length of stay (day)	13.500 (0.097)	9.998 (0.064)	<0.001
Operative time (h)	2.001 (0.017)	2.320 (0.147)	<0.001
Hospitalization expense (¥)	18240.099 (104.032)	17746.249 (77.067)	<0.001
Out of pocket expense (¥)	13068.592 (160.882)	13522.774 (103.574)	0.018
Medical insurance expense (¥)	5171.506 (149.313)	4223.475 (107.812)	<0.001
General medical service fee (¥)	1274.648 (19.500)	890.403 (8.459)	<0.001
General treatment operation fee (¥)	425.883 (3.952)	469.417 (3.139)	<0.001
Nursing fee (¥)	694.259 (5.468)	615.543 (3.585)	<0.001
Pathological diagnosis fee (¥)	917.366 (11.470)	1214.425 (13.335)	<0.001
Laboratory diagnostic fee (¥)	1839.740 (21.358)	1679.504 (12.243)	<0.001
Imaging diagnosis fee (¥)	1395.819 (23.223)	1496.950 (15.681)	<0.001
Surgical treatment fee (¥)	5344.472 (28.615)	5493.793 (17.973)	<0.001
Drug treatment fee (¥)	2799.103 (26.029)	2087.722 (17.978)	<0.001
Cost of antibacterial drugs (¥)	392.411 (13.075)	202.147 (9.041)	<0.001
Cost of disposable medical materials (¥)	2384.617 (23.771)	1919.360 (23.157)	<0.001
Admission route			0.014
Emergency clinic	239 (15.1%)	392 (12.5%)	
Outpatient service	1,348 (84.9%)	2,748 (81.5%)	
Surgical approach			0.014
Transabdominal	701 (44.2%)	710 (22.6%)	
Laparoscopic	715 (45.1%)	2,371 (75.5%)	
Transvaginal	171 (10.8%)	59 (1.9%)	
Surgical complications			<0.001
Yes	123 (7.8%)	153 (4.9%)	
No	1,464 (92.2%)	2,987 (95.1%)	
Nosocomial infection			<0.001
Yes	99 (6.2%)	92 (2.9%)	
No	1,488 (93.8%)	3,048 (98.2%)	
Emergency operation			<0.001
Yes	220 (13.9%)	282 (9.0%)	
No	1,367 (86.1%)	2,858 (91.0%)	
Return to the operating room for further surgery			0.572
Yes	6 (0.4%)	8 (0.3%)	
No	1,581 (99.6%)	3,132 (99.7%)	
Transfer out of ICU and then return to ICU			0.359
Yes	2 (0.1%)	10 (0.3%)	
No	1,585 (99.9%)	3,130 (99.7%)	
Blood transfusion			0.003
Blood transfusion volume ≥ 5 units or 1000 ml	12 (0.8%)	66 (2.1%)	
Blood transfusion volume < 5 units or 1000 ml	57 (3.6%)	115 (3.7%)	
No blood transfusion	1,518 (95.7%)	2,959 (94.2%)	
Diabetes			0.121
Yes	61 (3.8%)	94 (3.0%)	
No	1,526 (96.2%)	3,046 (97.0%)	
Hypertension			0.209
Yes	250 (15.8%)	451 (14.4%)	
No	1,337 (84.2%)	2,689 (84.6%)	
Thrombosis			0.675
Yes	30 (1.9%)	54 (1.7%)	
No	1,557 (98.1%)	3,086 (98.3%)	

BMI, body mass index; ICU, intensive care unit; HPV, Human papillomavirus.

### 3.2 The dynamic effects of interrupted time series analysis

**Figure 2:**
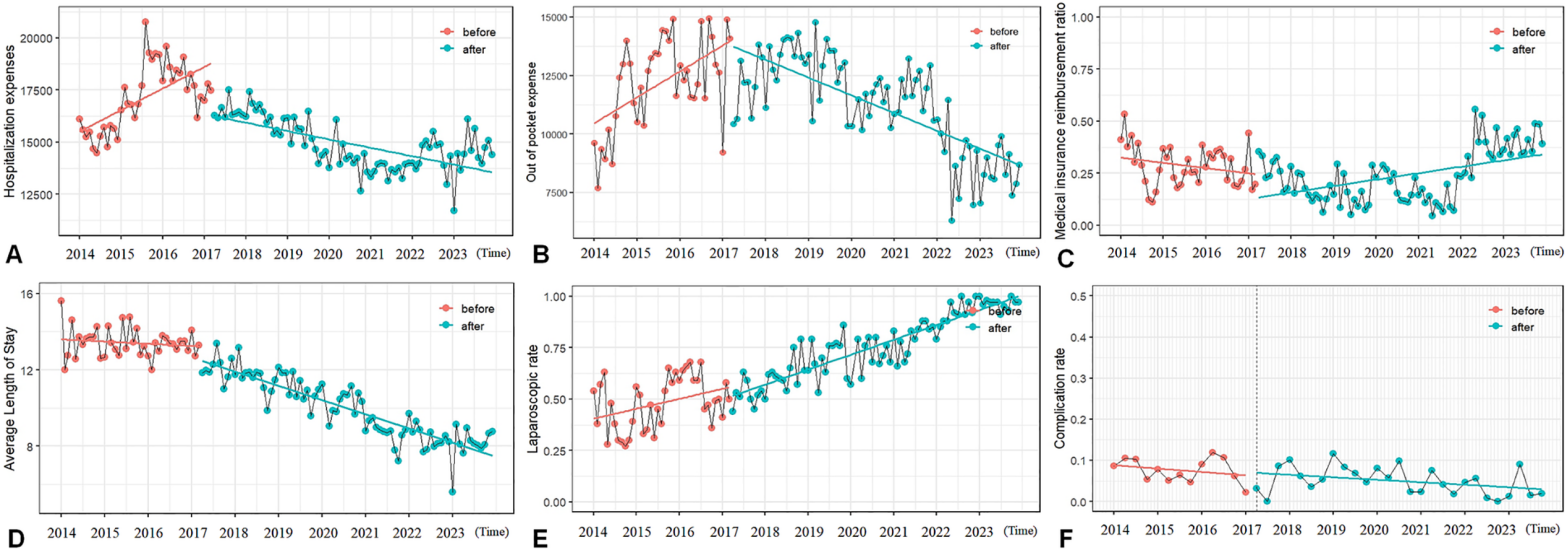
Analysis results of the interruption time series for all patients. **(A)** The hospitalization expenses. **(B)** The out-of-pocket expenses. **(C)** Medical insurance reimbursement ratio. **(D)** Average length of stay. **(E)** Laparoscopic proportion. **(F)** Incidence of complications.

The hospitalization expenses immediately decreased by 2265.51 ¥ (13.28%, *p* < 0.01) in the month of policy implementation. The upward trend of hospitalization expenses before the policy reversed to a continuous decrease, with a monthly decrease of 110.57 ¥ (*p* < 0.001; Figure 2A). The out of pocket expenses changed from an upward trend to a downward trend (monthly decrease of 178.65 ¥, *p* < 0.001, Figure 2B), and the medical insurance reimbursement ratio also changed from a decrease to an increase (monthly increase of 0.66%, *p* < 0.05, Figure 2C). The average length of hospital stay showed an immediate decrease (0.713 days, *p* < 0.01) and a sustained downward trend (monthly decrease of 0.053 days, *p* < 0.001, Figure 2D). No significant temporal changes were detected in the proportion of laparoscopic surgeries (Figure 2E) and the incidence of complications (Figure 2F) (*p* > 0.05).

**Figure 3:**
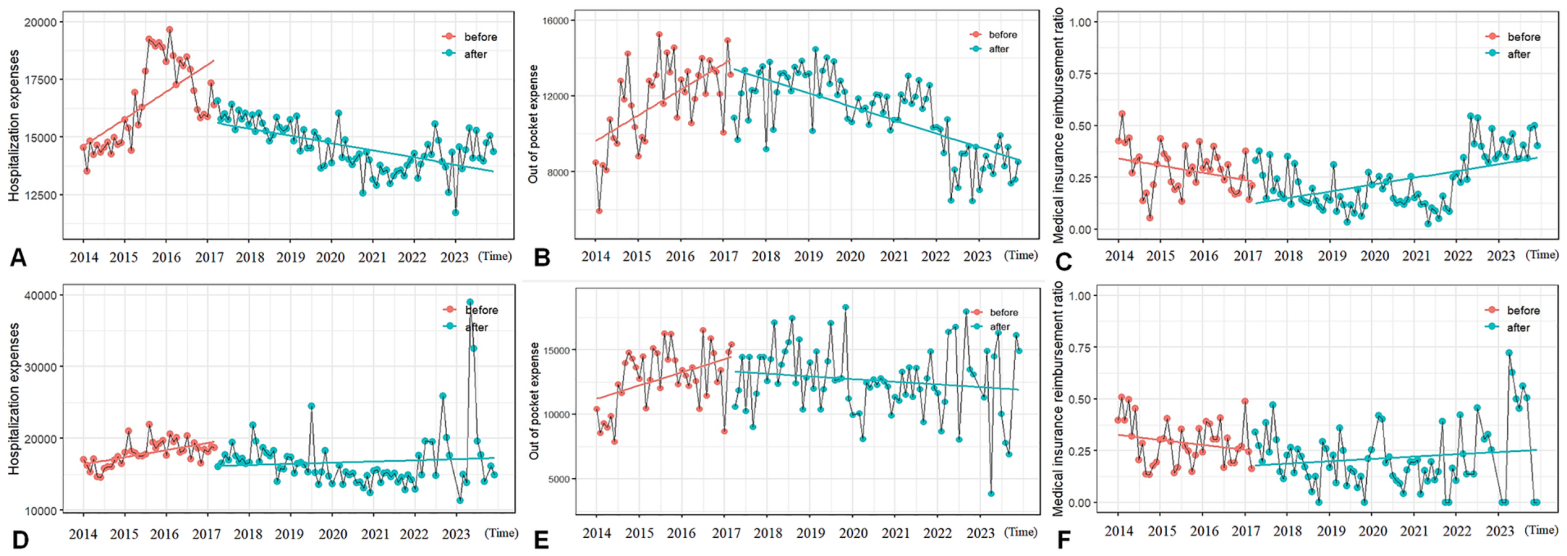
Analysis results of the interruption time series of subgroups of patients classified by surgical methods. **(A)** The hospitalization expenses of the laparoscopy group. **(B)** The out-of-pocket expenses of the laparoscopy group. **(C)** The medical insurance reimbursement ratio of the laparoscopy group. **(D)** The hospitalization expenses of the transabdominal group. **(E)** The out-of-pocket expenses of the transabdominal group. **(F)** The medical insurance reimbursement ratio of the transabdominal group.

The hospitalization expenses among women received laparoscopic surgeries showed an immediate decrease (1,908.91 ¥, *p* < 0.05) and a continuous decreasing trend (monthly decrease of 108.50 ¥, *p* < 0.001, Figure 3A). The out of pocket expenses had shown a continuous downward trend (monthly decrease of 197.43 ¥, p < 0.001, Figure 3B). The medical insurance reimbursement ratio showed a continuous upward trend (monthly increase of 0.71%, *p* = 0.012, Figure 3C). The abdominal surgery group only showed a decreasing trend in out of pocket expenses (monthly decrease of 103.81 ¥, *p* = 0.008, Figure 3E). There was no significant change in the hospitalization expenses (Figure 3D) and medical insurance reimbursement ratio (Figure 3F). No statistically significant effects were observed on all expense indicators in the vaginal surgery group.

### 3.3 Annual change trajectory of expenses

**Figure 4:**
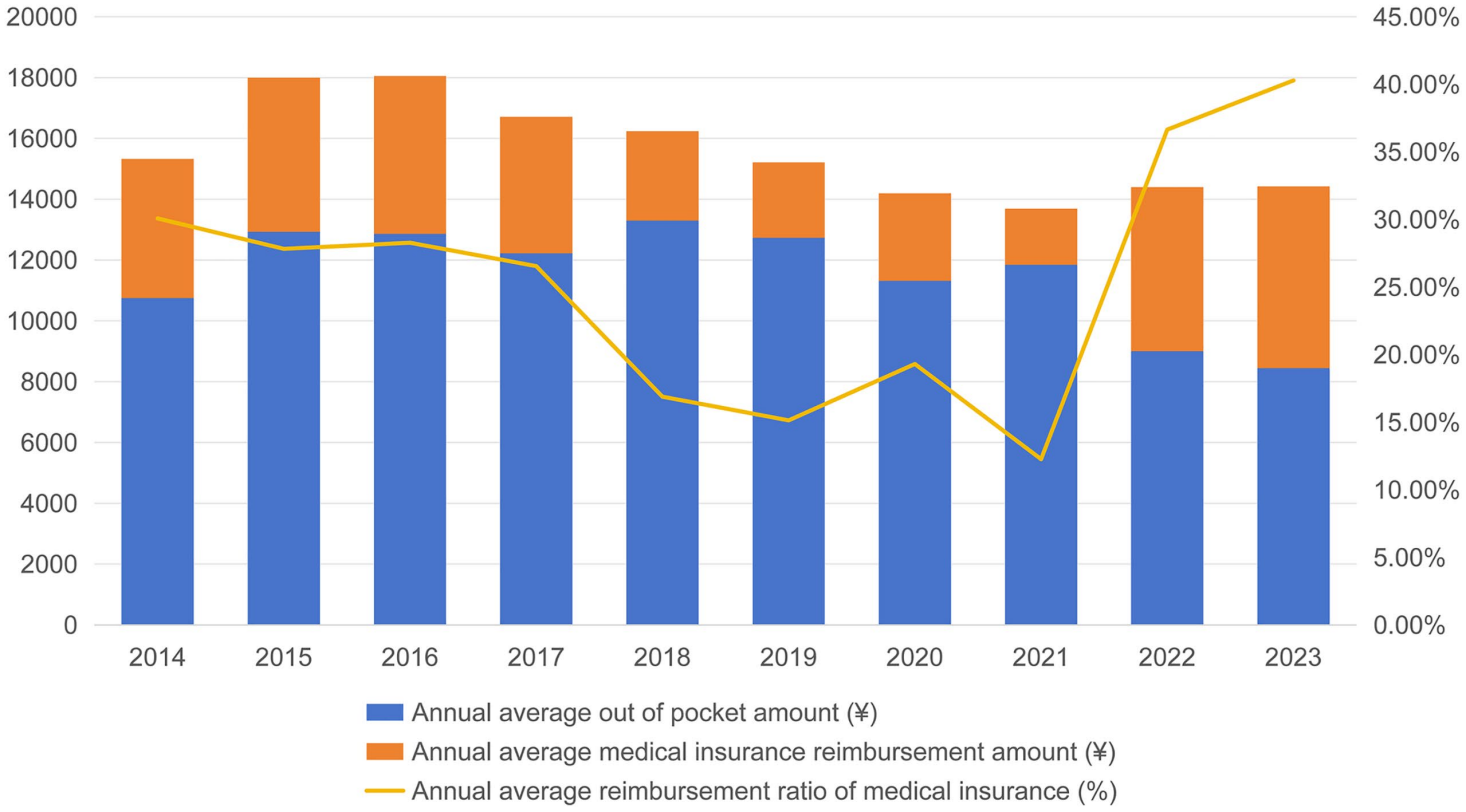
Annual trends of out of pocket expenses, medical insurance expense, and medical insurance reimbursement ratios.

The 10-year longitudinal follow-up (Figure 4) found that the out-of-pocket expenses of patients presented a “U-shaped” curve. In the early stage of the policy (2018–2021), the out-of-pocket expenses were higher than the baseline level, but then gradually decreased, reaching the lowest point in a decade (8,445.32 ¥) in 2023. After a decline in the initial stage of the policy, the medical insurance reimbursement ratio had continued to rise, reaching a historical peak of 40.29% in 2023, with the average annual medical insurance expense increasing to 5,976.23 ¥ in the same year (Tables 3, 4).

**Table 3:** Analysis results of interruption time series for all patients.

Variables	**Coefficient**	**Std. Error**	***t* value**	***P* value**
Hospitalization expenses (¥)				
β_0_: baseline level	15638.89	587.57	25.538	<0.001
β_1_: baseline slope	72.37	24.49	2.955	<0.01
β_2_: level change after policy	−2077.41	624.97	−3.324	<0.01
β_3_: slope change after policy	−107.85	27.08	−3.983	<0.001
Out of pocket expense (¥)				
β_0_: baseline level	10188.74	687.45	14.821	<0.001
β_1_: baseline slope	104.62	29.54	3.542	<0.001
β_2_: level change after policy	−593.06	790.37	−0.750	0.454575
β_3_: slope change after policy	−166.42	31.71	−5.248	<0.001
Medical insurance reimbursement ratio (%)				
β_0_: baseline level	0.371234	0.065676	5.653	<0.001
β_1_: baseline slope	−0.005064	0.002703	−1.873	0.0636
β_2_: level change after policy	−0.009201	0.067363	−0.137	0.8916
β_3_: slope change after policy	0.007127	0.003024	2.357	<0.05
Average length of stay (day)				
β_0_: baseline level	13.620684	0.254846	53.447	<0.001
β_1_: baseline slope	−0.009817	0.011099	−0.885	0.3783
β_2_: level change after policy	−0.712598	0.301683	−2.362	<0.05
β_3_: slope change after policy	−0.053139	0.011733	−4.529	<0.001
Laparoscopic proportion (%)				
β_0_: baseline level	0.405866	0.036971	10.978	<0.001
β_1_: baseline slope	0.003846	0.001599	2.406	<0.05
β_2_: level change after policy	−0.047115	0.043123	−1.093	0.2769
β_3_: slope change after policy	0.002305	0.001705	1.352	0.1791
Incidence of complications (%)				
β_0_: baseline level	0.0908837	0.0208108	4.367	0.000102
β_1_: baseline slope	−0.0021156	0.0025965	−0.815	0.420546
β_2_: level change after policy	0.0080870	0.0228304	0.354	0.725240
β_3_: slope change after policy	0.0005808	0.0027736	0.209	0.835324

**Table 4:** Analysis results of interruption sequences for each subgroup of patients classified according to surgical procedures.

Variables	**Coefficient**	**Std. Error**	***t* value**	***P* value**
Laparoscopic group				
Hospitalization expenses (¥)				
β_0_: baseline level	14,867.99	652.65	22.781	<0.001
β_1_: baseline slope	69.91	26.85	2.604	<0.05
β_2_: level change after policy	−1611.37	668.42	−2.411	<0.05
β_3_: slope change after policy	−102.14	30.05	−3.399	<0.001
Out of pocket expenses (¥)				
β_0_: baseline level	9,409.90	676.14	13.917	<0.001
β_1_: baseline slope	118.89	29.17	4.076	<0.001
β_2_: level change after policy	−612.18	784.70	−0.780	0.437
β_3_: slope change after policy	−178.77	31.19	−5.732	<0.001
Medical insurance reimbursement ratio (%)				
β_0_: baseline level	0.371942	0.061014	6.096	<0.001
β_1_: baseline slope	−0.004672	0.002566	−1.821	0.0713
β_2_: level change after policy	−0.049584	0.066480	−0.746	0.4573
β_3_: slope change after policy	0.007155	0.002813	2.543	<0.05
Abdominal group				
Hospitalization expenses (¥)				
β_0_: baseline level	16,376.99	1607.92	10.185	<0.001
β_1_: baseline slope	79.18	69.02	1.147	0.2538
β_2_: level change after policy	−3157.46	1862.37	−1.695	0.0928
β_3_: slope change after policy	−69.12	75.14	−0.920	0.3596
Out of pocket expenses (¥)				
β_0_: baseline level	11114.18	831.09	13.373	<0.001
β_1_: baseline slope	85.48	36.21	2.361	<0.05
β_2_: level change after policy	−1104.42	995.69	−1.109	0.26976
β_3_: slope change after policy	−103.81	38.68	−2.684	<0.01
Medical insurance reimbursement ratio (%)				
β_0_: baseline level	0.343635	0.066564	5.162	<0.001
β_1_: baseline slope	−0.002964	0.002857	−1.037	0.302
β_2_: level change after policy	−0.031321	0.077074	−0.406	0.685
β_3_: slope change after policy	0.003479	0.003111	1.118	0.266
Transvaginal group				
Hospitalization expenses (¥)				
β_0_: baseline level	14,475.36	1,299.70	11.137	<0.001
β_1_: baseline slope	73.97	59.51	1.243	0.218
β_2_: level change after policy	−1458.66	1,763.17	−0.827	0.411
β_3_: slope change after policy	−61.93	81.49	−0.760	0.450
Out of pocket expenses (¥)				
β_0_: baseline level	10,793.42	1,246.55	8.659	<0.001
β_1_: baseline slope	34.10	57.25	0.596	0.5532
β_2_: level change after policy	1,887.00	1,709.80	1.104	0.2734
β_3_: slope change after policy	−140.35	77.72	−1.806	0.0751
Medical insurance reimbursement ratio (%)				
β_0_: baseline level	0.237150	0.079700	2.976	<0.01
β_1_: baseline slope	0.001138	0.003659	0.311	0.75676
β_2_: level change after policy	−0.148729	0.109224	−1.362	0.17754
β_3_: slope change after policy	0.003389	0.004972	0.682	0.49761

## 4 Discussion

By employed an ITS design with a 10-year clinical data, this study, to our best knowledge, is the first study to systematically explore the short- and long-term effect after CP-based SDP policy which was implemented in China. Our results revealed significant effects and dynamic evolutions across structural resource allocation, service process adjustment, and clinical-economic outcomes.

Post-policy implementation, the proportion of laparoscopic surgeries surged from 45.1 to 75.5% (*p* < 0.001), becoming the dominant approach (20). This aligns with the global trend toward minimally invasive procedures, directly driving improvements in key process and outcome indicators: significant shortening of average length of stay (13.50 to 9.99 days, *p* < 0.001), and reductions in surgical complication rate (7.8 to 4.9%, *p* < 0.001) and nosocomial infection rate (6.2 to 2.9%, *p* < 0.001). This exemplifies Donabedian’s logic chain where “structural optimization drives process efficiency and outcome improvement” (21). The ITS model indicates that the adoption rate of laparoscopic surgery has been steadily increasing over the past decade. The growth rate post-policy (slope = 0.62%) is slightly higher than that before the policy (0.39%), but this acceleration does not reach statistical significance (*p* = 0.1791). This suggests that the popularity of minimally invasive surgery had already become an established long-term trend prior to the reform. The CP-SDP was not the sole driving factor, but rather provided an economic and regulatory environment conducive to this shift. By establishing fixed payment standards, the policy incentivized clinicians to favor laparoscopic surgery due to its superior recovery outcomes and lower long-term resource consumption, effectively aligning policy objectives with technological advancements. The 2.6% decrease in emergency admission rate (*p* = 0.014) indicates optimized outpatient referral mechanisms and improved resource allocation efficiency.

Findings strongly support Roemer’s prediction of “service intensity redistribution” under prepayment. A significant shift occurred from reducing therapeutic consumables to increasing diagnostic services: antibacterial drug costs dropped by 48.5%, disposable material costs by 19.5% (both *p* < 0.001), while pathological diagnosis fees rose by 32.4%, imaging diagnosis fees by 7.3%, general treatment operation fees by 10.2%, and surgical treatment fees by 2.8% (all *p* < 0.001). The decrease in the cost of disposable medical materials partly stems from the hospital’s optimization of the supply chain under the incentives of prepayment, including cost control for high-value consumables (e.g., ultrasonic scalpels) and the application of more cost-effective domestic alternatives. It should be noted that this study did not systematically collect intraoperative failure data for different brands/models of consumables. However, the concurrent significant reduction in postoperative complications and nosocomial infection rates indirectly suggests that this cost-containment strategy did not come at the expense of fundamental surgical safety and quality. The aforementioned adjustments reflects proactive optimization of diagnostic pathways by institutions under fixed prepayment—reducing high-variable-cost consumables (e.g., drugs, materials) while reinforcing diagnostic services critical for decision-making and quality management. This finding echoes the increased inspection cost ratio in Anhui’s SDP study (6), suggesting cross-regional universality.

Importantly, ITS analysis showed an immediate decrease in average length of stay at policy implementation (*p* < 0.01), with a sustained downward trend post-policy (*p* < 0.001). This dynamic trend synergized with the surge in laparoscopic surgeries to drive substantial reductions in total hospitalization duration, illustrating institutions’ inherent incentive to improve resource efficiency through rapid response and continuous improvement under prepayment. Additionally, the increase in elective surgery proportion (86.1 to 91.0%, *p* < 0.001) reflects standardized preoperative assessment under clinical pathway management, consistent with Zhang et al.’s report on “process standardization reducing resource waste” (7).

Clinically, significant improvements were observed: complication rate decreased from 7.8 to 4.9% (*p* < 0.001), and nosocomial infection rate from 6.2 to 2.9% (*p* < 0.001), with no significant changes in readmission indicators (reoperation, ICU readmission) or thrombosis rate (*p* > 0.05). These gains likely stem from laparoscopic surgery’s advantages in minimizing trauma and accelerating recovery, demonstrating structural optimization-driven outcomes. However, the proportion of massive blood transfusion increased from 0.8 to 2.1% (*p* = 0.003). The reasons for this change are not entirely clear. While theoretical concerns exist regarding potential changes in the use of hemostatic materials under cost containment, the significant decline in overall postoperative complication and infection rates, along with the stability of metrics like reoperation rate, indicates that the fundamental quality of perioperative safety management was maintained. The increased need for transfusion may reflect subtler shifts in surgical technical complexity not captured by the indicators collected in this study (e.g., specific fibroid size, location, preoperative anemia). This finding suggests that future research should incorporate more granular clinical variables to dissect the specific impacts of payment reform on intraoperative resource use and decision-making.

Economically, hospitalization expenses decreased by 2.7% (*p* < 0.001), achieving CP-based SDP’s core cost-control goal. Analysis showed a 18.3% reduction in medical insurance expenses (*p* < 0.001), while a simple comparison of means between the pre- and post-policy groups showed an increase of 3.5% in patient out of pocket expenses (*p* = 0.018). This short-term static finding aligns with Roemer’s law predicting potential initial cost-shifting under prepayment systems (12). This aligns with Zhang et al.’s (2024) findings in Fujian (7), highlighting potential risks to healthcare accessibility for low-income groups.

The dynamic perspective afforded by interrupted time series analysis reveals a more comprehensive long-term trajectory. The ITS model demonstrates a fundamental reversal in the trend of out-of-pocket expenses following policy implementation, shifting from a pre-policy increase to a significant post-policy decline (a decrease of 178.65 ¥ per month, *p* < 0.001). Further analysis of the annual cost trajectory reveals this pattern more clearly: out-of-pocket expenses exhibit a distinct “U-shaped” curve, remaining above the baseline in the initial years following policy implementation (approximately from 2018 to 2021), before entering a sustained decline phase in 2021. We analyze that this subsequent decline is driven by multiple factors: First, 2021 was a pivotal year for the comprehensive expansion of Diagnosis-Related Groups (DRG) and Diagnosis-Intervention Groups (DIP) payment reforms in Fujian. According to the 14th Five-Year Plan for Universal Medical Security in Fujian Province, these reforms intensified the incentives for hospitals to optimize cost structures beyond the initial SDP framework. Second, the national and provincial Volume-Based Procurement (VBP) policies for medical consumables and drugs were fully implemented around 2021, leading to a sharp decrease in the cost of high-value consumabless (22, 23). Finally, the provincial medical insurance administration adjusted reimbursement policies in 2021, further increasing the fund’s coverage ratio for inpatient expenses (24). Consequently, out of pocket expenses reached their lowest point in the decade by 2023. Therefore, a complete interpretation must encompass two dimensions: the short-term financial pressure on patients during the initial policy adjustment and the more substantive, long-term reduction in financial burden achieved through combined policy and market mechanisms. This dynamic indicates that after an initial transition, the CP-SDP policy fostered a sustainable equilibrium characterized by concurrent reductions in both insurance fund expenditures and patients’ long-term out of pocket expenses.

Furthermore, ITS analysis of hospitalization expenses showed an immediate reduction of 2,265.51 ¥ at policy implementation (*p* < 0.01), likely due to the rapid compression of non-core costs such as high-value consumables. More importantly, the pre-policy upward trend was reversed into a sustained downward trajectory post-policy (*p* < 0.001), reflecting continuous efficiency improvements through process re-engineering. Mirroring this pattern, the pre-policy rising trend in out of pocket expenses also reversed into a significant decline (*p* < 0.001), culminating in the lowest annual out of pocket amount in 2023. This shift signals that cost-containment pressure was ultimately absorbed by providers, benefiting patients—a phenomenon similar to the “cost burden rebalancing” observed in Shanghai’s DRG pilot (25). Simultaneously, the medical insurance reimbursement ratio reversed from a downward to an upward trend (*p* < 0.05), peaking in 2023, which indicates enhanced financial protection for patients even as total hospitalization costs fell. Collectively, these long-term trends demonstrate that the policy, through continuous regulatory refinement and the scale effects of laparoscopic technology, successfully optimized cost structures, effectively alleviated the patient financial burden, and established a sustainable positive cycle.

The subgroup ITS analysis further clarified the heterogeneity of different surgical methods in terms of cost dynamics. The laparoscopic surgery group demonstrated the most significant cost control effect. The hospitalization expenses not only decreased immediately by 1,908.91 ¥ (*p* < 0.05), but also showed a continuous downward trend (*p* < 0.001); The downward trend of out of pocket expenses and the upward trend of the medical insurance reimbursement ratio were equally significant (*p* < 0.05). This highlights laparoscopic surgery’s cost-effectiveness and scale advantages as the policy’s primary carrier. In contrast, abdominal surgery showed only a slight downward trend in out-of-pocket expenses (*p* = 0.0084), with non-significant changes in hospitalization expenses and reimbursement, indicating limited cost-control space for traditional approaches. Vaginal surgery showed no significant expense dynamics, directly addressing the practical challenge of “whether SDP should adopt differentiated pricing for different approaches” and emphasizing new technology diffusion (e.g., laparoscopy) in policy effectiveness.

This study systematically elucidates the transmission mechanism of SDP driving process efficiency improvement and ultimately improving clinical and economic outcomes through structural optimization and service intensity redistribution, by integrating two theoretical frameworks. The specific advantages are reflected in the following four aspects. Firstly, this study innovatively combined Donabedian’s three-dimensional quality theory with Roemer’s law to construct a comprehensive evaluation model covering resource allocation, service intensity adjustment, and cost-quality balance. This model constructed a systematic assessment framework for the quality of health services from three dimensions: structure, process and result. Meanwhile, we utilized Roemer’s Law of health service supply to reveal the correlation between supply quantity and utilization rate. And we applied the above two, respectively, to form complementary support from the perspective of the logic of quality composition and the law of supply behavior, jointly enhancing the reliability of the derivation conclusion (21). Secondly, through 10-year ITS analysis, the dynamic heterogeneity of policy effects was accurately quantified, and for the first time, the long-term optimization path of the ratio of patients’ out-of-pocket expenses to medical insurance reimbursement was revealed. Again, a stratified analysis was conducted on the three surgical methods of laparoscopy, laparotomy and transvaginal surgery. The “immediate and continuous” cost control effect of the laparoscopy group was quantified for the first time, providing an empirical basis for the specific payment standard of the surgical methods. Finally, covering the data of the first 3 years and the last 6 years of the policy implementation, the dynamic trajectory of the policy effect was fully presented. It was clarified that the clinical quality indicators decreased significantly after the policy implementation. In the context of the global transition toward DRG/DIP payment models, this study underscores the enduring value of CP-SDP as a standardized management instrument. Current national and provincial guidelines in China emphasize the construction of a multi-mode composite payment system, wherein SDP continues to function alongside DRG for specific surgical procedures with high path predictability. Our 10-year longitudinal data provide a robust empirical benchmark for ‘clinical pathway standardization’ within the DRG framework. Specifically, the findings illustrate how prospective payment mechanisms can catalyze technological diffusion (e.g., the surge in laparoscopic procedures) and structural cost optimization. These insights offer a replicable paradigm for refining DRG weighting systems and ensuring that payment reforms incentivize quality-driven innovation rather than merely reducing service intensity.

This study has some limitations. Firstly, the data originated from a tertiary grade-A obstetrics and gynecology specialized hospital. Specialized hospitals typically possess superior clinical pathway management capabilities and resources compared to general hospitals, which may overestimate the CP-SDP policy’s effectiveness and limit generalizability to less specialized settings. Additionally, institutional practice patterns, patient demographics, and regional insurance policies may introduce regional bias. Secondly, as a retrospective study, its analysis relies on historical management data, and there may be information bias and unmeasured confounding factors affecting the attribution of the results. Although ITS design is a powerful quasi-experimental method for evaluating policy effects, multi-center studies are still needed to verify the wide applicability of this finding. Therefore, in the future, the team plans to conduct a multi-center study to expand the sample size and cover patients from different regions and types of hospitals. At the same time, it will strictly standardize the data collection process to reduce selection bias and data quality issues, so as to more comprehensively and accurately assess the impact of the single-disease cost control policy on myomectomy in different medical environments.

## 5 Conclusion

This study demonstrates that CP-based SDP effectively optimizes hysterectomy practices for uterine fibroids by driving minimally invasive surgery adoption and service structure adjustment, achieving concurrent cost control and quality improvement. The policy significantly reversed the upward trend in patient out-of-pocket burdens and improved insurance reimbursement rates long-term, validating its sustainability. These findings provide a robust evidence base for refining medical insurance payment reforms, particularly in surgical specialties.

## Data Availability

The raw data supporting the conclusions of this article will be made available by the authors, without undue reservation.
